# The Relationship Between Emotion Processing and Flexor Afferent Responses in Upper Limbs

**DOI:** 10.3390/s26020557

**Published:** 2026-01-14

**Authors:** Gianluca Isoardo, Rita B. Ardito, Stefano Ciullo, Elena Fontana, Ilaria Stura, Giuseppe Migliaretti, Paolo Titolo, Enrico Matteoni, Andrea Calvo, Valeria Fonzo, Federica Laino, Mauro Adenzato

**Affiliations:** 1 SC Neurologia 1U, Hospital “Città della Salute e della Scienza di Torino”, 10126 Turin, Italy; 2 Department of Psychology, University of Turin, 10124 Turin, Italy; 3 Department Neuroscience “Rita Levi Montalcini”, University of Turin, 10126 Turin, Italy; 4 Department of Public Health and Paediatric Sciences, University of Torino, 10124 Torino, Italy; 5 UOD Reconstructive Microsurgery, Department of Orthopedics & Traumatology, Hospital “Città della Salute e della Scienza di Torino”, 10126 Turin, Italy; 6 SC Psichiatria SPDC, Hospital “Città della Salute e della Scienza di Torino”, 10126 Turin, Italy; 7 Unit of Pain Management and Palliative Care, Department of Anesthesia, Intensive Care and Emergency, Hospital “Città della Salute e della Scienza di Torino”, 10126 Turin, Italy

**Keywords:** neurophysiology, emotion, flexor reflexes, biomedical sensing, electrodiagnostic system, nerve conduction studies, quantitative sensory testing (QST), stimulation threshold (mA), cutaneous silent period (CSP), cutaneomuscular reflex (CMR)

## Abstract

Background: This study investigates the influence of emotional processing on flexor reflex responses in the upper limbs, focusing on cutaneomuscular reflexes (CMRs) and the cutaneous silent period (CSP) in patients with chronic neuropathic pain. The modulation of motor reflexes by emotions remains unclear. Methods: Fifty-one patients with chronic upper limb neuropathic pain (carpal tunnel syndrome, other neuropathies, post-burn hypertrophic scars) and twenty healthy controls underwent standardized electrodiagnostic signal acquisition. Neurophysiological assessments (CMRs, CSP, standard nerve conduction tests) and psychological evaluations (anxiety, depression, emotion processing) were conducted. Neurophysiological signal acquisition included median and ulnar nerve conduction studies recorded with an electrodiagnostic system (48 kHz sampling rate; 30–3000 Hz bandpass). CSP and CMRs were recorded from the abductor pollicis brevis using surface electrodes (bipolar belly–tendon montage) and were evoked by electrical stimulation delivered through ring electrodes, with individualized perceptual-threshold calibration. Statistical analyses examined correlations between neurophysiological and psychological measures. Results: Patients showed significantly longer duration and higher intensity of CMRs and CSP than controls (*p* < 0.01). CMR and CSP durations correlated positively with anxiety, depression, and alexithymia scores, and negatively with facial emotion recognition. General Linear Model analyses indicated these relations were mediated by tactile and pain perception thresholds. Conclusions: The findings support that spinal reflex responses in the upper limbs are modulated by emotional and cognitive-affective processes, especially in chronic pain contexts. This highlights the complex interaction between emotion regulation and motor control in neuropathic pain conditions.

## 1. Introduction

Sherrington, in his 1910 pivotal work on the flexion reflex of the limb, defined the protective nature of the flexion reflex and its stereotypical pattern of muscle activation in response to a stimulus applied to a receptive field. Notably, he described a combination of vocalization, retracting the lips, opening the mouth, and turning of the head sideways and backward, together with flexion of the limb in the decerebrate cat, and coined the term “pseudoaffective reflex” to define a combination of reflex movements in preparation for defence [1]. Sherrington’s view that reflexes are stereotyped and fixed motor responses to a stimulus applied to a receptive field has changed, and reflexes are now regarded as flexible responses integrated by centrally generated motor commands to produce movements suitable for an ever-changing environment [2].

Within the group of reflex responses mediated by the so-called flexor reflex afferents, withdrawal reflexes are considered nociceptive responses [3,4], while cutaneomuscular reflexes (CMRs) are regarded as responses elicited by non-nociceptive stimuli and involved in fine motor control [3,4,5,6]. Although withdrawal reflexes are considered spinal circuitry-mediated and stereotyped responses [3], their amplitude can be modulated by emotional stimuli depending on the positive or negative valence of these stimuli [7,8,9]; for instance, amplitude of the withdrawal reflex recorded from the biceps femoris can be increased after phobic stimulation [7]. In the upper limb, the withdrawal response to noxious stimuli differs in the arm, forearm, and intrinsic hand muscles: in the hand muscles, a suppression of ongoing electromyographic activity, known as the cutaneous silent period (CSP), is prominent, whereas in the arm and forearm, there is a contraction of relaxed muscles [10]. Consistent with previous reports correlating withdrawal reflexes and emotions, CSP has been found to be related to anxiety scores on the Hospital Anxiety and Depression Scale [11]. Observation of another individual’s painful stimulation of the dorsum of the hand negatively modulates motor-evoked potentials recorded from the first dorsal interosseus in the observer [12]. This provides a clue to the link between unconscious modulation of motor output and conscious feelings.

CMRs coexist with CSP during high-intensity electrical stimulation of the fingers, and both CMRs and CSP are topographically organized. In thenar muscles, CMR may include a late inhibitory component (I2), sometimes preceded by an excitatory component (E2) [3,4,6,13,14]. Both E2 and probably I2 are mediated by a transcortical circuitry [13,14]. The amplitude of the E2 component superimposed on CSP is modulated by the proximity of the stimulated hand to the face, that is, the peripersonal space [14]. The increase in E2 with the hand near the face parallels that of the somatosensory blink reflex, which is modulated by empathy, anxiety, and the organization of the mental representation of attachment in childhood [15]. Thus, it is conceivable that flexor reflex afferent responses, including both withdrawal responses and CMRs, may be modulated by higher-order cognitive and emotional processes to better shape behaviour in response to the environment. In the present study, we correlated the metrics of intrapersonal and interpersonal emotion regulation, mood, and anxiety with both CMRs and CSP recorded from the abductor pollicis brevis in patients with neuropathic pain in the upper limb and healthy controls. To better define the mediators of possible correlations between CMR/CSP and psychological findings, we also took into account the psychophysical evaluation of Aβ and Aδ sensory fibres involved in CMR and CSP generation [3,4,5,6,12].

## 2. Materials and Methods

### 2.1. Patients

The study was approved by the Ethics Committee of the Città della Salute e della Scienza di Torino Hospital. Patients and controls provided informed consent to participate in the study. We enrolled patients with chronic (lasting more than 3 months) peripheral neuropathic pain [16,17,18] involving at least one hand. The patients cohort included individuals with carpal tunnel syndrome (CTS), brachial plexopathy, painful cervical radiculopathy, ulnar neuropathy at the elbow, or post-burn hypertrophic scars (PBHSs), and the diagnosis of each disease was made as previously described [19,20,21]. All patients underwent a full clinical evaluation as described [6,19,20,21], including pinprick, touch, and position sense assessments of both upper limbs. In addition to evaluating pinprick and touch sense, the pain sites were also assessed for signs of allodynia in response to brushing, as part of the DN4 questionnaire [22]. Self-reported mean pain intensity in the week before examination was graded on an 11-point numerical rating scale (NRS), with scores ranging from 0 (no pain) to 10 (worst possible pain) [23].

### 2.2. Neurophysiological and Psychophysiological Evaluation

Sensory and motor nerve conduction studies of the median and ulnar nerves were performed as described [6,19,20,21], using a commercially available electrodiagnostic equipment (Viking Quest, Carefusion, Middleton, WI, USA). The sampling rate was 48 kHz, with a bandpass of 30 to 3000 Hz. CSP and CMRs of the abductor pollicis brevis (APB) were recorded with surface electrodes in a bipolar belly–tendon montage. To elicit both CSP and CMRs in patients and controls, electric shocks were delivered to the index finger while subjects performed an isometric contraction at maximum force against a resistance [6,19]. To maintain constant contraction strength, audio feedback was provided to patients and controls. CSP and CMRs obtained during maximal contraction tend to be shorter in duration [5]; nonetheless, we performed CSP and CMR evaluation during maximal strength contraction to avoid eliciting the late excitatory component of CMRs in APB [5]. Stimulation was delivered through ring electrodes (Carefusion, Middleton, WI, USA), with the cathode placed at the index proximal interphalangeal joint. CSP was obtained after stimulation at an intensity eight times the perceptual threshold for an electric shock, while CMR was obtained at an intensity twice the perceptual threshold [6]. This threshold was determined separately for each hand by slowly increasing the intensity of stimulation delivered at 1 Hz until the patients perceived a sensation of a non-painful electric shock. To evaluate CSP, off-line rectified electromyographic activity was averaged over eight trials for each hand. To evaluate CMR, electromyographic activity was off-line rectified, and each of the ten traces that showed suppression of activity lasting more than 10 ms was included for analysis. To avoid habituation, each trial was performed at least 60 s after the previous trial. Onset and offset of CSP and CMRs were defined by the same author (G.I.) by visual inspection as the beginning of an abrupt decrease and recovery of electromyographic activity, as previously described [6,19]. This method of detection has been used in previous studies [6,19]. Before performing these studies, two experienced neurophysiologists separately evaluated the onset and offset of CSP and CMRs in patients and controls, with fair agreement between evaluations (k: 0.8, unpublished data).

All patients and controls underwent quantitative sensory testing (QST) evaluation of cold detection threshold (CDT) and heat pain detection threshold (HDT) at the dorsum of the hand and the palmar surface of the index and little fingers, and of vibration detection threshold (VDT) at the index and little fingers. Details on the QST procedure are provided elsewhere [6,19,20,21].

#### Psychological, Health Quality, and Social Support Evaluation

The following psychological evaluations were performed in all patients and controls: Toronto Alexithymia Scale (TAS-20) [24,25] for alexithymia, Beck Depression Inventory-II (BDI-II) [26] for depression, the Y form of the State-Trait Anxiety Inventory (STAI-Y) for anxiety symptoms [27], the Reading the Mind in the Eyes task (RME) [28] for affective theory of mind, the Ekman 60 Faces Test (EK-60) [29] for facial emotion recognition, and the Empathy Quotient [30] for empathy.

The 12-item General Health Questionnaire (GHQ-12) [31] was used to assess levels of psychological distress, and the Multidimensional Scale of Perceived Social Support (MSPSS) [32] was used to assess social support.

### 2.3. Statistical Analysis

The results are presented as mean ± standard deviation (SD) for continuous variables, and as absolute and relative frequencies for categorical variables. The normality of the distribution of continuous parameters was assessed using the Kolmogorov–Smirnov test. QST parameters that were not normally distributed were log-transformed for analysis with parametric inferential methods [20]. The z-scores of the patients at each site were calculated as follows:

(log/ln patient value − mean log/ln healthy controls value)/SD log/ln healthy controls value

Comparisons were performed using the *t*-test and ANOVA for normally distributed variables, or the Wilcoxon and Kruskal–Wallis tests for non-normally distributed variables.

Correlations were analyzed by estimating the parametric Pearson correlation coefficient (*r*). General Linear Models (GLMs) were used to test the dependence of CMRs and CSP on psychological evaluations, QST, and other parameters. Group, educational age, and achievements were considered as possible confounding factors; if significant, analyses are presented divided by these variables. Multiple comparison adjustments were made where necessary.

In all analyses, *p*-values < 0.05 were considered statistically significant. Statistical analysis was carried out using SAS^®^ Statistics Software v. 9.4.

## 3. Results

### 3.1. Demographic, Neurophysiological, and Psychophysical Findings

We enrolled 51 patients and 20 healthy controls. Data from all patients and controls were included in previously published studies [6,20,21]. In the patient group, 34 had CTS, 10 had PBHS, and 7 had other peripheral neurological diseases (ONDs) causing upper limb pain. Demographic data are shown in Table 1.

**Figure 1:**
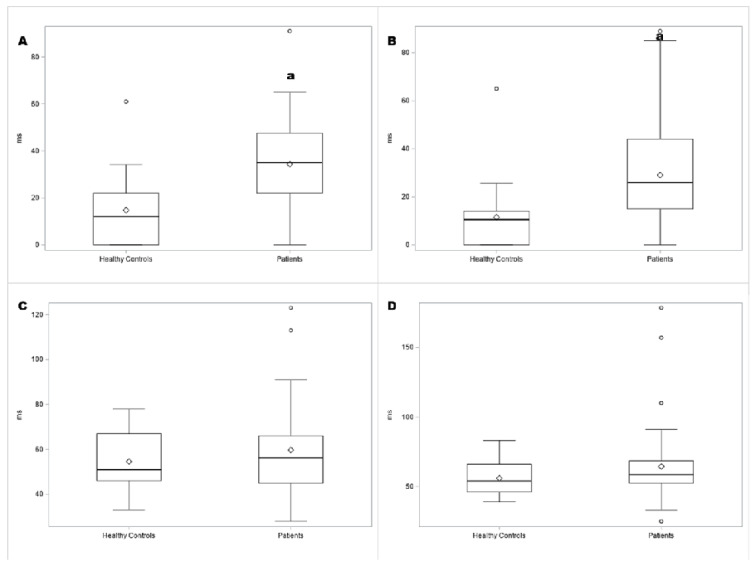
Box plots comparing patients and healthy controls (HC) for (**A**) right CMR, (**B**) left CMR, (**C**) right CSP, and (**D**) left CSP. The vertical axis indicates the duration of suppression of electromyographic activity in milliseconds (ms). ms: milliseconds. ^a^ *p* < 0.01 versus healthy controls.

**Figure 2:**
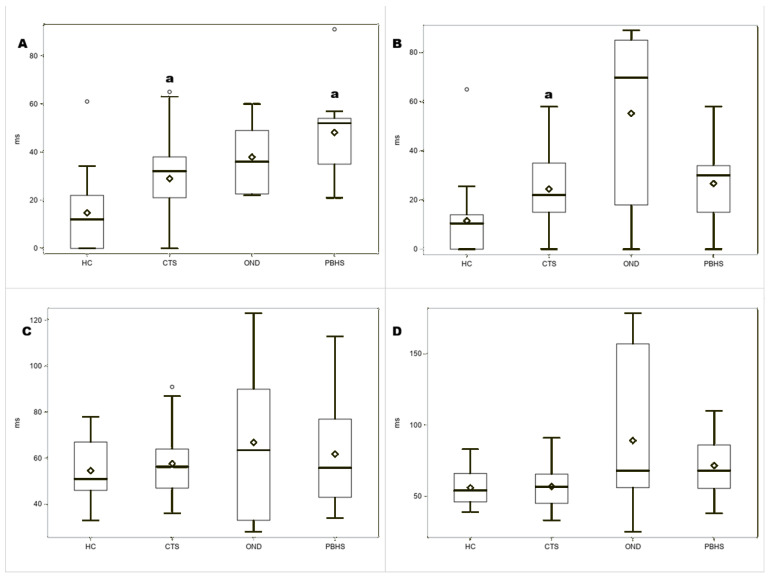
Box plots comparing patients with carpal tunnel syndrome (CTS), other neurological diseases (ONDs), post-burn hypertrophic scars (PBHSs), and healthy controls (HCs) for (**A**) right CMR, (**B**) left CMR, (**C**) right CSP, and (**D**) left CSP. The vertical axis indicates the duration of suppression of electromyographic activity in milliseconds (ms). ms: milliseconds; Diamond shape refers to mean. ^a^ *p* < 0.01 versus healthy controls.

**Figure 3:**
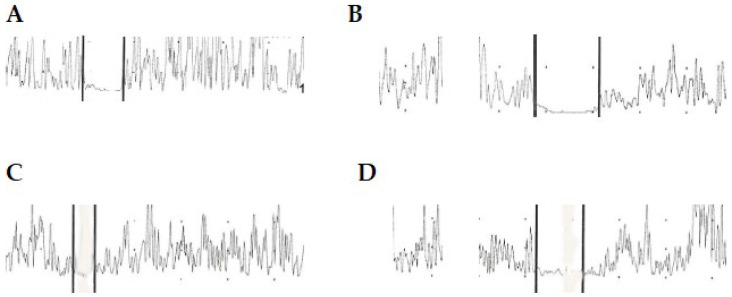
Examples of CSP and CMRs from the right abductor pollicis brevis in healthy controls and patients with neuropathic pain. (**A**) CSP from a healthy control. Stimulation was set at 50 milliampere (mA), duration was 50 milliseconds (ms). (**B**) CSP from a patient with a post-burn hypertrophic scar involving the right hand. Stimulation was set at 65.6 mA, duration was 70 ms. (**C**) CMR from the same healthy control. Stimulation was set at 12.5 mA, duration was 23 ms. (**D**) CMR from the same patient. Stimulation was set at 16.4 mA, duration was 50 ms (Vertical lines refers to onset and offset of electromyographic activity suppression).

As previously reported [20,21], there was no difference in sex or age distribution between patients and controls, but patients had fewer years of education than controls. Findings from nerve conduction studies and QST evaluations are shown in Table A1 and Table A2 (Appendix A). CSP and CMR findings are shown in Table 2. Box plots showing the duration of CMRs and CSP in patients and healthy controls, as well as among CTS, OND, and PBHS, are provided in Figure 1 and Figure 2. Examples of CMRs and CSP in patients and healthy controls are shown in Figure 3.

Median nerve sensory conduction velocity and median and ulnar amplitudes of sensory action potentials were lower in patients than in controls. A significant difference between patients and controls was evident for ln-transformed VDT at all evaluated sites and for log-transformed CDT at all sites, except the right dorsum and right little finger. No significant differences were observed in sensory and motor nerve conduction or QST findings, among CTS, PBHSs, and ONDs.

Bilaterally, CMR duration and the intensity of electric shock required to elicit CMRs and CSP were significantly higher in patients than in healthy controls, as shown in Figures. No significant differences in CSP and CMR parameters were found among CTS, PBHSs, and ONDs. CMR duration was higher than in healthy controls and on the right hand in PBHSs, as shown in Figure 2A,B.

### 3.2. Psychological Findings

Results of psychological evaluations are summarized in Table 3. Patients had higher BDI-II, TAS-20 overall score, TAS-20 F1 score, TAS-20 F3 score, and lower overall EK-60 and EK-60 subscore for fear than healthy controls. No differences were observed in psychological metrics among CTS, ONDs, and PBHSs, as previously described [20,21].

### 3.3. Correlation Between CSP, CMR, and Psychological Findings

Duration of right CMR correlated with STAI-Y1 (r = 0.44, *p* = 0.001), STAI-Y2 (r = 0.43, *p* = 0.03), TAS-20 overall (r = 0.46, *p* = 0.001), TAS-20 F1 subscore (r = 0.53, *p* < 0.0001), EK-60 overall score (r = −0.30, *p* = 0.005), and EK-60 subscore for sadness (r = −0.41, *p* = 0.01). Bilaterally, duration of CMR correlated with BDI-II (right r = 0.54, *p* < 0.0001; left r = 0.29, *p* = 0.01), and EK-60 subscore for sadness (right r = −0.38, *p* = 0.002; left r = −0.3, *p* = 0.01).

**Figure A1:**
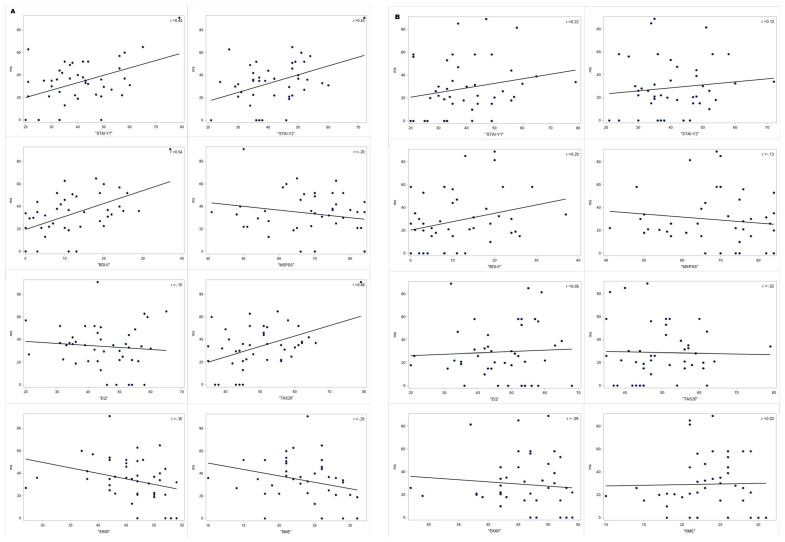

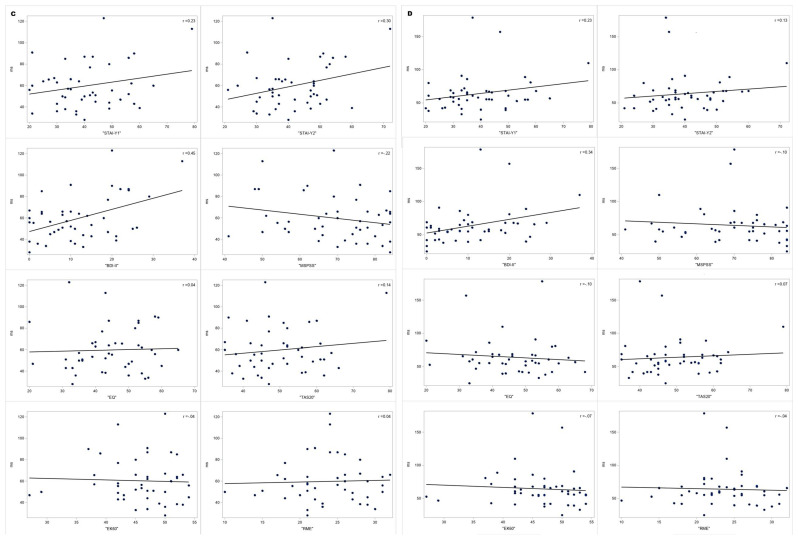
Scatterplots showing relationship between psychological findings in patients with neuropathic pain and duration of right CMR (**A**), duration of left CMR (**B**), duration of left CSP (**C**) and duration of right CSP (**D**).

Duration of CSP correlated bilaterally with BDI-II (right r = 0.34, *p* = 0.002; left r = 0.45, *p* = 0.008), and on the right side with TAS-20 F1 score (right r = 0.31, *p* = 0.01). Scatterplots showing the relationship between psychological findings and the duration of CMA and CSP are shown in the Figure A1.

### 3.4. General Linear Model (GLM)

When GLM analysis was performed to investigate the interaction among psychological findings and CMR and CSP parameters, the relationship between left CMR and CSP duration and BDI-II was mediated by STAI-Y1 (CMR: b = 0.6, standard error = 0.02, *p* = 0.02; CSP: b = 0.06, standard error = 0.02, *p* = 0.02) and STAI-Y2 (CMR: b = −0.09, standard error = 0.03, *p* = 0.01; CSP: b = −0.09, standard error = 0.03, *p* = 0.01). Right-hand CSP duration was also related to years of education (b = 1.5, standard error = 0.52, *p* = 0.005).

When GLM analysis of the relationship between CMR and CSP parameters with psychological findings was performed including QST results, the correlation between the duration of left CMR and BDI-II was entirely mediated by VDT z-score at the index finger (b = −0.6, standard error = 0.18, *p* = 0.0007) and little finger (b = 0.99, standard error = 0.38, *p* = 0.01). In contrast, the relationship between right CMR duration and STAI-Y2 was mediated by HPT z-score at the index finger (b = 0.1, standard error = 0.05, *p* = 0.02). Right-hand CSP duration is affected by the interaction between BDI-II and years of education (b = 1.7, standard error = 0.47, *p* < 0.001). The GLM did not identify other factors influencing the relationship between psychological findings and CMR/CSP parameters.

## 4. Discussion

The results reported here demonstrated a relationship between the duration of CMR and CSP and metrics of state and trait anxiety, depression, emotion recognition (EK-60), and emotion regulation (TAS-20). GLM showed that the relationship between left CMR and depression depends on VDT z-scores at the index and little fingers, and that the relationship between right CMR and trait anxiety is mediated by the HPT z-score at the index finger. Years of education only affected the relationship between right CSP and BDI-II.

The relationship between left CMR duration and depression is mediated by vibratory perception in the left index and little fingers. These results are consistent with our previous findings on the relationship between vibration perception in the left index finger and alexithymia, which in turn is a risk factor for the development of major depression [33]. In fact, CMR is mediated by Aß sensory fibres [3,4,5,6,13]; therefore, the relationship between left CMR duration and depression appears to be related to the function of Aß afferents from the left-hand fingers. The relationship between right CMR duration and trait anxiety is also consistent with the relationship between right-hand HPT z-scores and state anxiety observed in our previous study [20]. In contrast, GLM did not show any influence of QST findings on the relationship between right CMR duration and EK-60 total score, nor any influence of years of education on the relationship between CMR duration and psychological features. It is noteworthy that in our previous study [21], only the EK-60 score for surprise correlated with vibration perception at the left index, while neither the overall EK-60 score nor its subscores correlated with QST parameters or QST findings on the right hand. To our knowledge, this is the first study to comprehensively evaluate the relationship among CMRs/withdrawal reflexes and intrapersonal and interpersonal emotion processing. The correlation between CSP and anxiety [11], modulation of withdrawal reflex amplitude in response to phobic stimulation [7], and inhibition of intrinsic hand muscle motor-evoked potentials after cortical stimulation by observation of another person’s pain [12] further support a relationship among mood, anxiety, emotion processing, and motor control. These findings, along with our observations, appear to contrast with other animal model studies describing two different pathways for reflexive and affective behaviour after noxious stimulation [34]. However, Mas-related G protein-coupled receptor D-positive spinal neurons are involved in reflexive responses in healthy mice, but their activation elicits affective behaviour in mice with chronic pain [34]. This observation suggests that pain may modulate the activity of spinal neurons, thus allowing the expression of either simple reflexive or more complex affective behaviour. The so-called “emotional motor system”, responsible for behaviour in response to emotion-inducing stimuli [35,36], encompasses the periaqueductal grey and the locus coeruleus (LC). The LC can modulate withdrawal reflexes in rats [37] via its spinally projecting neurons [38]. Furthermore, the LC firing pattern (tonic or phasic) can modulate behavioural modes ranging from distractibility to task-oriented behaviour elicited by decision processing and salient sensory stimuli [39]. LC cortical projections include the anterior cingulate cortex and motor cortex [39]. Emotion-regulating neural circuits, including the amygdala and anterior cingulate cortex, can modulate the brainstem nuclei involved in the emotional motor system. Layer 5 neurons in the prelimbic area in mice (corresponding to area 32 in humans [40]) project to the ventrolateral periaqueductal grey, which in turn projects to the LC [41]. This circuit is inhibited by projections from the basolateral amygdala. Therefore, activation of the basolateral amygdala influences avoidance behaviour by modulating the prelimbic cortex/ventrolateral periaqueductal grey circuit, thus reducing noradrenergic e serotoninergic inputs to the spinal cord [41]. These observations further suggest that forelimb and brainstem structures controlling withdrawal reflexes also control more complex behaviour; therefore, it is conceivable that modulation of both withdrawal reflexes and CMRs is part of the response to emotion-inducing stimuli, particularly in the presence of chronic pain. Our study highlighted the correlations between both CMRs and withdrawal reflexes in the upper limbs and metrics of intrapersonal and interpersonal emotion regulation. Only some of the correlations were influenced by sensory perception or years of education; thus, the correlation of right-hand CMR (a component of optimal motor control [6]) with alexithymia and emotion recognition in others (evaluated by EK-60) seems to suggest a direct interaction between emotion recognition and motor control.

Eliciting withdrawal reflexes in humans activates of the anterior cingulate cortex and deactivates the posterior cingulate cortex [42]. As these cortical areas are also strongly involved in emotion processing, a functional overlap between nocifensive reflex circuits and emotional regulation is plausible. Furthermore, various aspects of social cognition [43], and emotions in particular, are considered primary reinforcers that guide goal-directed actions in the environment [44]. Within this framework, it is therefore conceivable that components of the motor infrastructure, such as CMR, which is involved in fine hand movements [3,5,6], as well as withdrawal nocifensive reflexes [3,4], may be modulated by emotion-regulation processes and mood.

### Limitations of the Study

This study includes patients with neuropathic pain involving the upper limb, so the relationship between emotion regulation, CMRs, and withdrawal reflexes could be influenced by pain itself. Further studies on different neurological and psychiatric patient groups could elucidate whether CMRs and withdrawal reflexes are related to emotion processing independently. In addition, studies on patients with non-neuropathic pain (nociceptive and primary chronic pain) could clarify the effect of pain itself versus its pathophysiology on the relationship between CMRs/withdrawal reflexes and emotion processing.

## Figures and Tables

**Table 1:** Demographic and clinical features of patients and controls.

	All Patients	Healthy Control
Age	53.6 ± 13.4	44.4 ± 13.4
Male/Female	18/33	5/15
Years of education	10.9 ± 3.3 ^a^	14.2 ± 3.9
NRS	6.1 ± 2.4	-
DN4	5.4 ± 2.3	-
CTS	34	-
PBHS	10	-
ONP	7	-

DN4: DN4 questionnaire; CTS: carpal tunnel syndrome; NRS: 11-point numerical rating scale; ONP: other neuropathic pain; PBHS: post-burn hypertrophic scars. ^a^ *p* < 0.01 versus healthy controls.

**Table 2:** Summary of CMR and CSP results.

	Patients	Healthy Controls
CMR		
Latency to onset (ms)		
Right	92.9 ± 16.6	95.4 ± 14.9
Left	89.5 ± 18.9	97.0 ± 22.1
Duration (ms)		
Right	34.3 ± 19.3 ^a^	14.7 ± 15.7
Left	29 ± 23.5 ^a^	11.5 ± 15.6
Latency to offset (ms)		
Right	131.6 ± 14.6 ^b^	118.6 ± 16.1
Left	125.8 ± 20.3	114.3 ± 11.8
Threshold (mA)		
Right	18.1 ± 9 ^a^	11.5 ± 3.8
Left	19.7 ± 10.8 ^a^	10.9 ± 3.4
CSP		
Latency to onset (ms)		
Right	71.5 ± 11.2	70.8 ± 8.2
Left	71.4 ± 12.1	70.2 ± 6.5
Duration (ms)		
Right	59.7 ± 20.7	54.6 ± 13.5
Left	64.3 ± 27.2	55.9 ± 12.8
Latency to offset (ms)		
Right	131.2 ± 17.2	125.4 ± 11.9
Left	135.7 ± 24.7	126.1 ± 12.1

CMR: cutaneomuscular reflexes; CSP: cutaneous silent period; mA; milliampere; ms: milliseconds. ^a^ *p* < 0.01 versus healthy controls. ^b^ *p* < 0.05 versus healthy controls.

**Table 3:** Summary of psychological evaluation results.

	Patients	Healthy Controls
BDI-II	11.3 ± 8.9 ^b^	5.8 ± 4.8
STAI-Y1	39.9 ± 12.5	34.6 ± 7.3
STAI-Y2	41.1 ±10.1	39.3 ± 7.9
GHQ12	2.7 ± 3.1	1.6 ± 2.5
MSPSS		
Overall	69.7 ± 11.6	68.5 ± 12.5
Family	23.5 ± 4.6	21.8 ± 6.9
Friends	20.9 ± 6.2	22.7 ± 4
Others	24.8 ± 3.6	23.4 ± 5.1
EQ	45.6 ± 10.8	46.4 ± 8.3
TAS20		
Overall	49.9 ± 9.2 ^a^	43.1 ± 6.9
F1	17 ± 5.9 ^b^	13.3 ± 3.7
F2	12.8 ± 3.9	12.9 ± 3.7
F3	19.9 ± 3.7 ^a^	16.8 ± 4.5
Ek-60		
Overall	46.2 ± 5.6 ^b^	49.5 ± 4.1
Happiness	9.7 ± 0.7	9.9 ± 0.2
Surprise	9 ± 1	9.3 ± 1
Anger	7 ± 1.6	7.5 ± 1.5
Disgust	8.4 ± 1.6	9 ± 1.1
Sadness	7.1 ± 1.5	7.5 ± 1.7
Fear	5.2 ± 2.3 ^a^	6.4 ± 1.9
RME	23.6 ± 4.7	25 ± 4.5

BDI-II: Beck Depression Inventory-II; EK-60: Ekman 60 Faces Test; EQ: empathy quotient; GHQ12: General Health Questionnaire; MSPSS: Multidimensional Scale of Perceived Social Support; RME: Reading the Mind in the Eyes task; STAI: State-Trait Anxiety Inventory; TAS-20: Toronto Alexithymia Scale. ^a^ *p* < 0.01 versus healthy controls. ^b^ *p* < 0.05 versus healthy controls.

## Data Availability

The data in this article may be provided upon request.

## Abbreviations

The following abbreviations are used in this manuscript:

DN4	DN4 questionnaire
CTS	Carpal tunnel syndrome
NRS	11-point numerical rating scale
ONP	Other neuropathic pain
PBHS	Post-burn hypertrophic scars
CMR	Cutaneomuscular reflexes
CSP	Cutaneous silent period
BDI-II	Beck Depression Inventory-II
EK-60	Ekman 60 Faces Test
EQ	Empathy quotient
GHQ12	General Health Questionnaire
MSPSS	Multidimensional Scale of Perceived Social Support
RME	Reading the Mind in the Eyes task
STAI	State-Trait Anxiety Inventory
TAS-20	Toronto Alexithymia Scale

## Appendix A

**Table A1:** Summary of motor and sensitive nerve conduction study results.

					Median					
	MCV (m/s)		SCV (m/s)		CMAP Amplitude (mV)		SAP Amplitude (mV)		Latency (ms)	
	Right	Left	Right	Left	Right	Left	Right	Left	Right	Left
Median Nerve										
All patients	52.7 ± 4.8 ^b^	52.8 ± 5.2 ^b^	46.8 ± 8.1 ^a^	46.9 ± 9.1 ^a^	9.9 ± 4.3	10.2 ± 6	23.6 ± 15.6 ^a^	29.6 ± 21.5 ^a^	4.3 ± 1.1 ^a^	4.1 ± 1 ^a^
Healthy controls	58.3 ± 6.6	60 ± 5.3	59.1 ± 7.7	62.5 ± 7.5	9.6 ± 4	10.4 ± 5.9	52.7. ± 21.3	58 ± 21.3	3.2 ± 0.3	3.1 ± 0.3
Ulnar Nerve										
All patients	59.1 ± 6.4	60.1 ± 6.5	57.4 ± 6.5	57 ± 7.8	8.1 ± 3	7.7 ± 2.8	30.2 ± 16.4 ^b^	33.8 ± 17.5 ^b^	2.6 ± 0.5	2.7 ± 0.6
Healthy controls	59 ± 6.5	58 ± 6.2	57.8 ± 6	58 ± 6	9.9 ± 4.1	7.4 ± 3.8	53.7 ± 23	52.7 ± 23.5	2.7 ± 0.3	3 ± 0.5

CMAP: Compound Muscle Action Potential; m = metres; MCV: Motor Conduction Velocity; SCV: Sensor Conduction Velocity; SAP: Sensor Action Potential; ms: milliseconds; mV = milliVolts; s = seconds. ^a^ *p* < 0.0001 versus healthy controls. ^b^ *p* < 0.01 versus healthy controls.

**Table A2:** Summary of quantitative sensory testing results.

	Patients	Healthy Controls
CDT		
Dorsum		
Right	1.491 ± 0.02 (−2.65 ± 6.42)	1.499 ± 0.003
Left	1.420 ± 0.410 ^a^ (−28.6 ± 145.9)	1.500 ± 0.003
Index		
Right	1.467 ± 0.05 ^b^ (−4.29 ± 9.59)	1.497 ± 0.004
Left	1.427 ± 0.375 ^b^ (−8.43 ± 42.29)	1.496 ± 0.01
Little finger		
Right	1.472 ± 0.044 (−3.22 ± 14.09)	1.487 ± 0.013
Left	1.383 ± 0.413 ^a^ (−9.58 ± 25.90)	1.486 ± 0.023
HPT		
Dorsum		
Right	1.633 ± 0.04 (−0.15 ± 1.03)	1.635 ± 0.039
Left	1.629 ± 0.039 (−0.29 ± 1.07)	1.634 ± 0.037
Index		
Right	1.663 ± 0.026 (0.17 ± 0.79)	1.658 ± 0.035
Left	1.635 ± 0.139 (−0.48 ± 3.99)	1.655 ± 0.034
Little finger		
Right	1.654 ± 0.031 (−0.03 ± 0.89)	1.659 ± 0.038
Left	1.655 ± 0.033 (−0.13 ± 0.93)	1.660 ± 0.039
VDT		
Index		
Right	−0.313 ± 0.931 ^b^ (0.70 ± 1.13)	−0.972 ± 0.966
Left	−0.291 ± 1.319 ^b^ (0.92 ± 1.70)	−0.995 ± 0.664
Little finger		
Right	−0.225 ± 0.855 ^a^ (0.81 ± 1.92)	−1.054 ± 0.651
Left	−0.265 ± 1.076 ^a^ (0.83 ± 1.20)	−0.852 ± 0.764

CDT: cold detection threshold; HPT: heat pain threshold; VDT: vibration detection threshold. Results of CDT and HPT evaluations are log-transformed. Results of VDT evaluation are ln-transformed. Z-scores are indicated in parentheses. ^a^ *p* < 0.01 versus healthy controls. ^b^ *p* < 0.05 versus healthy controls.
